# Development and Characterization of AZ91 Magnesium Alloy Reinforced with Ti Wires

**DOI:** 10.3390/ma18112517

**Published:** 2025-05-27

**Authors:** Wojciech Wyrwa, Adrianna Filipiak-Kaczmarek, Anna Nikodem

**Affiliations:** 1 Department of Lightweight Elements Engineering, Foundry and Automation, Faculty of Mechanical Engineering, Wrocław University of Science and Technology, 27 Wybrzeże Stanisława Wyspiańskiego St., 50-370 Wrocław, Poland; adrianna.filipiak@pwr.edu.pl; 2 Department of Mechanics, Materials and Biomedical Engineering, Wrocław University of Science and Technology, 27 Wybrzeże Stanisława Wyspiańskiego St., 50-370 Wrocław, Poland; anna.nikodem@pwr.edu.pl

**Keywords:** AZ91, composite, titanium reinforcement, SBF

## Abstract

Lightweight metals are increasingly used in biomedical engineering, and can be found in orthopaedics (screws, implants), stomatology, cardiology (stents) and as scaffolds. Magnesium alloys have a low density (1.74 g/cm^3^), which is very close to that of bone (1.75 g/cm^3^), as well as high biocompatibility, and are biodegradable. Unfortunately, their disadvantage is their low resistance to corrosion in the human body, which further causes deterioration of mechanical and physical properties. Improvement of these properties can be achieved by making the composite on a magnesium matrix—depending on the reinforcement added, the required properties can be obtained. This paper presents the results of a study on the extrusion of a magnesium matrix composite with titanium (Ti) reinforcement. The study included three-point bending tests, from which it is clear that the introduction of Ti reinforcement improves the bending strength of the specimens. In addition, the samples were immersed in SBF (simulated body fluid) for 1, 2, 4, 8, 12 and 24 h to determine the degradation of the Mg–Ti composite.

## 1. Introduction

Due to their biocompatibility and biodegradability properties, magnesium alloys could be used in biomedical engineering—especially in orthopaedics as implants. But in this application, their disadvantages are low mechanical properties and low resistance to corrosion in the human body. A solution to these problems could be a magnesium matrix with titanium reinforcement.

Several studies have shown that strengthening the magnesium matrix with titanium components can improve the mechanical properties of the composite. It is worth mentioning that among metal particles, which could be used as a reinforcement for the magnesium matrix, titanium particles are considered to be an ideal reinforcing phase [1]. It is argued that titanium shares the same typical hexagonal crystal structure (HCP) as magnesium and these metals will not react to form any intermetallic compounds [2,3,4,5,6,7,8]. Apart from this, the addition of Ti increases wear resistance without compromising mechanical strength and ductility [9]. Nonetheless, in the case of using powders, the key and difficult issue is dispersing the particles of the reinforcements uniformly in the matrix. Nowadays, the following methods are used: stir casting, friction stir processing, and powder metallurgy [10,11,12]. Kumar et al. [9] prepared AZ91–Ti composites using powder metallurgy techniques and conducted a study to determine the influence of this reinforcement on wear resistance. The results showed that the addition of this metal improves abrasion due to the scratch and wear properties of the titanium reinforcement. Moreover, it was observed that the best results were obtained for samples where 6% titanium was added to AZ91. It should be emphasized that a further increase in titanium content caused a slight decrease in wear resistance. The authors argue that this was due to the easy removal of agglomerated Ti particles and the formation of cavities.

Luo et al. [13] conducted a study on a magnesium matrix composite that was reinforced with TC4 to determine structural and mechanical properties. During a tensile test, it was observed that the AZ91-5 wt% composite had a higher tensile strength. The authors argue this result by the presence of a strong connection between the magnesium matrix and the titanium reinforcement, which is possible through the formation of coherent interfacial bonding of Al_3_Ti/Mg_21_(Zn, Al)_17_ and semi-coherent interfacial bonding of Mg_21_ (Zn, Al)_17_/Mg. The tensile strength and elongation started to decrease when the TC4 particle content was above 5 wt% and the difference between 10% and 15% reinforcement content was not significant. The explanation for this could be the following: the existence of TC4 affects the interaction of dislocations, thereby improving the work hardening effect of the composites [14]. Nevertheless, the increase in reinforcement particles results in the grain boundaries weakening to an excessive degree, which results in decreased strength and plasticity as a consequence.

Pu et al. [1] investigated the influence of titanium particles (5 wt%) on the microstructure and mechanical properties of TiP/AZ91 composites prepared via powder metallurgy. It was concluded that the strength and ductility of the composite were improved. The ultimate tensile strength increased significantly from 163 MPa to 221 MPa and the elongation increased from 3.5% to 10.9%. The powder metallurgy technique used ensured uniform dispersion of titanium particles in the magnesium matrix and the Mg_17_Al_12_ content was reduced. The authors indicate that the strengthening mechanism of the TiP/AZ91 composites mostly includes dislocation strengthening, dispersion strengthening and load transfer strengthening. The toughening mechanism of the investigated composites is as follows: the titanium particles are deformable and the addition of them could introduce many twins and dislocations to coordinate plastic deformation and improve plasticity.

Nevertheless, reinforcing the magnesium matrix with titanium mesh is not popular yet, and is most often used in oral implantology. It is characterized by high strength and stiffness, good plasticity and low thickness. Additionally, titanium mesh has excellent biological properties such as good tissue compatibility, low electrical conductivity and corrosion resistance. However, its mechanical properties are highly dependent on its thickness and porosity [15].

It should be emphasized that for biomedical applications, the thickness of the titanium mesh is also important for another reason. Many studies have suggested that this parameter may affect the total amount of new bone formation. Bai et al. [16] designed a titanium membrane (Ti-Mesh) for alveolar bone defects. According to their findings, when the thickness of the mesh increases, its bending strength also increases. A study showed that 100–200 µm is the ideal thickness of titanium mesh to reconstruct a large number of bone defects [15,16].

Among the implants applied in medicine, there are permanent and temporary ones. Permanent implants serve as a replacement for natural bone by restoring its function. In general, the second group acts as a support until bone tissue heals. Magnesium-based alloys can be used as materials for temporary biomedical implants because of their natural biodegradability in the physiological environment. Unfortunately, common applications of them are still limited due to their uncontrollable degradation rate in the physiological environment. A high concentration of chloride anions makes it more aggressive compared to the typical aqueous solution [17,18].

Developed engineering solutions aim to respond to requirements concerning mechanical properties, biocompatibility, and degradation rate.

Modifications to the composition of magnesium-based alloys include the incorporation of some body nutrient elements such as Zn, Ca, and Mn. Investigations have also been carried out on Mg-Al, Mg-Zr and Mg-RE (rare-earth elements) based alloys; for example, Y, Gd, Nd, and Ce. Such operations aim to improve mechanical and chemical properties. The degradation rate here depends on the particular composition [18].

To enhance the degradation resistance of Mg-based alloys, surface modification is employed. Bio-inert and bio-active coatings should be noted here.

Bio-inert coatings are obtained using the following technologies: electrochemical deposition, plasma spray, micro-arc oxidation, physical vapour deposition, chemical vapour deposition, ion implantation, conversion coatings, and laser treatments. However, according to the name, coatings created by these techniques are non-biodegradable [18].

Among bioactive coatings, it can be listed electrodeposited coatings, bio-mimetic coatings, and chemical conversion coatings. For instance, the addition of bioactive elements such as Ca and P in the coating by micro-arc oxidation technique, results in a composition more similar to bone and a reduction in the degradation rate [19].

In the work [18], innovative future prospects of Mg-based alloys have been listed, which included:

- Nano-phased alloy. For instance, nano-grained alloys made up of Ti-6Al-4V, cobalt-chromium alloys, stainless steels, etc. and some nanoceramic coatings of alumina, nano-hydroxyapatite, etc. have shown enhanced cell adhesion and proliferation relative to traditional implant materials [20,21].
- Development of functionalized Mg-bone implants, which can have additional purpose. For instance, the delivery of medicament at a specific site of an organ [22].
- Fabrication of Mg-based implants with tunable interfaces. Modification of the wettability to develop a hydrophobic surface can decrease the degradation rate. It can be realized through chemical etching, application of laser, and surface lithography [23,24].
- Hybrid coatings are developed by combining both organic and inorganic components. For instance, in the work [25], Singh et al. realized a TiO_2_ –HAp-PCL (polycaprolactone) based hybrid coating, where TiO_2_ –HAp was the inorganic part responsible for high adhesion strength and improving osteogenesis. PCL was an organic part responsible for improving corrosion resistance.
- Advancement of additive manufacturing technology for implant production. For instance, laser-based additive manufacturing can fabricate customized implants with effective efficiency and offer process flexibility [26].

This paper aimed to prepare the magnesium matrix composite with titanium mesh via pressure infiltration and to assess the influence of this modification on the exploitable properties of the material. The research used magnesium alloy AZ91, whose density (1.85 g/cm^3^) is similar to that of human cortical bone (1.75 g/cm^3^) and has a Young’s modulus of 44 GPa. However, the main disadvantage of the AZ91 alloy as a biomaterial is its susceptibility to corrosion in the environment of the human body [27,28]. In this study, the AZ91 alloy matrix was reinforced with two and three layers of Ti mesh. The microstructure, mechanical properties and behaviour during strength tests of these materials were examined. Additionally, ageing tests in an SBF solution were carried out.

## 2. Materials and Methods

### 2.1. Materials

The commercial AZ91 alloy (Prometal, Trzebina, Poland) was used to prepare the Mg matrix composite due to its good mechanical properties and castability. The chemical composition of the AZ91 alloy is listed in Table 1.

**Figure 1:**
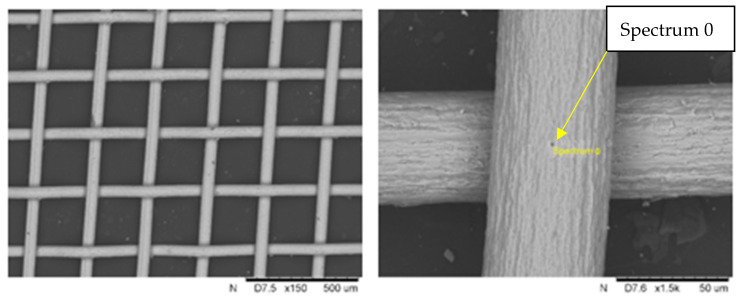
Titanium mesh, Alfa Aesar, Spectrum 0: 100 wt% Ti. The scale value denoted in the figures by ‘um’ represents micrometers (µm).

The titanium mesh—Alfa Aesar titanium gauze, 100 mesh woven from 0.05 mm dia wire—(Thermo Fisher Scientific, Warsaw, Poland) was used as a reinforcement for the composite. Its chemical composition is presented in Table 2. Figure 1 provides the SEM image of it.

The point analysis confirmed the chemical composition and purity of the fibres, which have a regularly wrinkled surface, without porosity or cracks. The diameter does not differ from that stated (50 µm), and it seems that at the points of contact of perpendicular fibres, a connection was created via micro-welding.

In this study, three types of specimens: AZ91 alone, AZ91 with two layers of titanium mesh and AZ91 with three layers of titanium mesh, were used. The AZ91/Ti mesh composite was produced with a squeeze casting method. It is worth mentioning that this process is classified as pressure infiltration and can be used to manufacture metal matrix composites where molten metal fills a porous reinforcement [29]. In this method, infiltration and solidification can be controlled by pressure [30]. Basically, the process consists of pouring molten metal into a preheated die produced from special alloy steel. When the filling is complete, a piston usually applies pressure to the molten metal head. This helps to ensure that the metal feeds the solidifying casting, minimizing shrinkage and micro-shrinkage porosity [31,32]. This method enables the production of machine parts that are almost ready to use. The process parameters that were selected to obtain the best possible composite material properties are temperature of the molten metal and die, position and structure of the preform, infiltration pressure and flow velocity.

To prepare the composite, the Ti fibre preform was initially made, then placed in the central part of the die. A metal holder was used to position and hold the mesh layers. The preform and the casting die were preheated to 600 °C and 300 °C correspondingly. The AZ91 alloy was superheated to 700 °C and then poured onto the preform with a compaction pressure of 150 MPa. The process was conducted in such a way that the metal stream came from all sides. The squeeze casting process was used because of simple process parameter control, good wettability of the reinforcement by the liquid metal, and better metallurgical quality of matrix alloys due to s-olidification under pressure.

Beam specimens of the as-cast materials in dimensions 40 mm in length, 5 mm in width, and 3 mm in thickness were machined for different testing and investigations.

### 2.2. Accelerated Aging Tests

The magnesium beams reinforced with titanium mesh were subjected to accelerated ageing tests in SBF—simulated body fluid—(ABO, Gdańsk, Poland) for 24 h. The SBF has a composition similar to that of human blood (Table 3). The samples were immersed in SBF and placed in containers in the Pol–Eko SLW 53 TOP+ drying oven maintained at a temperature of 36.6 °C, which was intended to simulate the environment of the human body. Short-term intervals were used for the experiment, during which the mass of the samples and the pH of the SBF were measured at intervals of 1, 2, 4, 8, 12 and 24 h. After each interval, additional SEM photos were taken.

### 2.3. Material Characteristics and Mechanical Tests

Material analysis was carried out by SEM using a Hitachi TM3000 scanning electron microscope with the EDS/EDX system (Hitachi High-Technologies Corporation, Tokyo, Japan). The microstructure analysis was performed using a SkyScan 1172 microcomputed tomography scanner (Bruker, Kontich, Belgium) with an X-ray source set at 89 kV and 112 μA. Sample imaging was performed with a spatial resolution of 3 μm, using Al + Cu filters, a rotation step of 0.2° and a full rotation range of 360°. The scanning parameters of the tested samples are listed in Table 4.

Each composite sample was subjected to strength tests. In this case, a three-point bending test was performed. The three-point bending tests of the AZ91 + Ti mesh composite were conducted using a Tinius Olsen H25KT testing machine (TINIUS OLSEN Ltd., Surrey, UK) The test was performed at a speed of 2 mm/min and a maximum force of 50 N.

## 3. Results

### 3.1. Bending Strength Properties with Fracture Analysis

**Figure 2:**
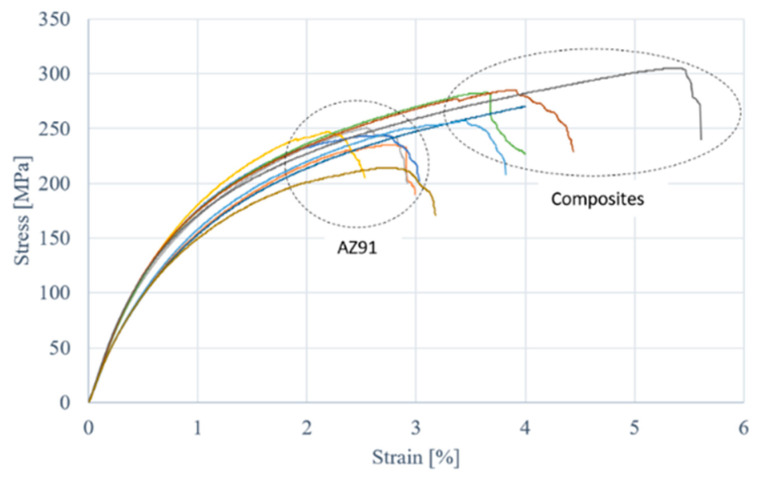
Bending strength for AZ91 and composite samples of AZ91 with two layers of Ti mesh.

The mechanical properties of the prepared samples were studied via a three-point bending test, whose results are shown in Table 5 and Figure 2. The flexural strength for the AZ91 samples ranged from 235 to 250 MPa. In the case of samples reinforced with two titanium mesh layers, strength increased by approximately 11%, ranging from 257 to 283 MPa. Similarly, for samples reinforced with three titanium mesh layers, an additional increase in flexural strength was observed compared to the previous two groups of samples—meaning by ca. 9% compared to composite with two mesh layers and by ca. 22% compared to the pure alloy.

**Figure 3:**
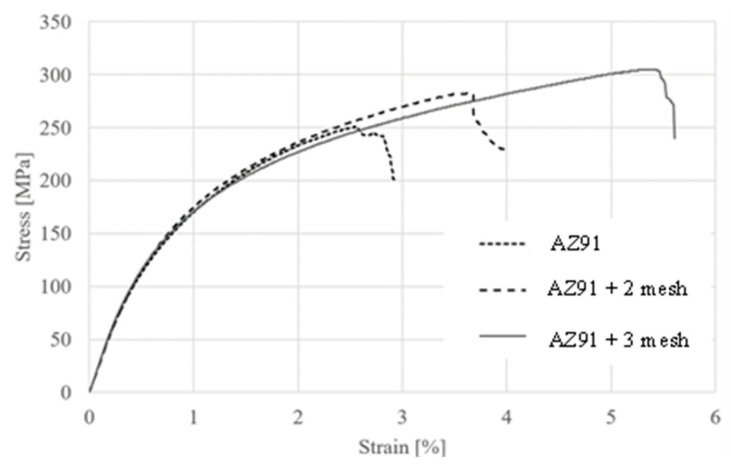
Bending test results for AZ91 matrix samples, and reinforced composite with Ti mesh.

Figure 3 shows the most representative curves for pure AZ91, AZ91 with two mesh layers and AZ91 with three mesh layers. For instance, using three layers of Ti mesh allows for high deformation, exceeding 5% at a destruction force of approximately 300 MPa. Increasing the amount of reinforcement led to an increase in the flexural strength of the samples. When comparing the bending curves for all three types of samples, it was observed that the curves had a similar shape—the only difference was the range of deformation for each sample. The most significant difference was noted between the pure AZ91 sample and the AZ91 sample reinforced with two titanium mesh layers. In this case, the increase in strength was substantial (Figure 3). The volume fraction and contribution to load transfer of the Ti fibres are very small. The cross-section of the beam is 15 mm^2^, in which one mesh layer with 20 wires covers ca. 0.039 mm^2^. This corresponds to 0.5% in the case of the sample with two mesh layers. Comparing this share with the 12% increase in strength, it can be concluded that the strengthening effect is significant. Microscopic observations indicate that titanium fibres mainly transfer tensile stress. Although their UTS is 240 MPa, there must be other strengthening mechanisms, e.g., grain refinement or microcrack arrest.

However, the trend is different for the Young’s modulus. The highest estimated Young’s modulus value was observed for the composite reinforced with two layers of titanium mesh (27–28 GPa). The addition of another mesh layer worsened the estimated value of this parameter (23–24 GPa). Similar results were obtained in the study [35] by Hassan et al. where they investigated the effect of copper reinforcement on the strength properties of AZ91. When 3.9% Cu was added to AZ91, an increase in Young’s modulus was observed. However, with higher reinforcement content, the value of Young’s modulus decreased. This is due to the high modulus of reinforcement (i.e., 129.8 GPa for Cu [35]) and uniform distribution of reinforcement with good interfacial integrity.

Bones have a bending strength of 50–120 MPa, depending on the type of bone. Therefore, any material used for implants should have similar mechanical properties to those of bone. The test results clearly indicate that the material can be used as an implant, due to its mechanical properties, which are similar to those of bone, especially Young’s modulus [36].

**Figure 4:**
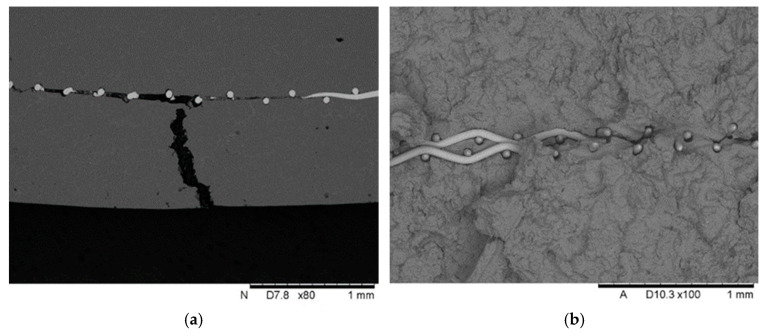
Crack of sample at deflection alongside the Ti reinforcement (**a**), fracture in perpendicular direction for sample with 2 mesh layers (**b**).

As a result of the three-point bending tests, the samples were damaged and cracked. Figure 4 shows that the crack starts at the edge of the sample, reaches the titanium mesh and then spreads along the reinforcement. In other studies [37], it was observed that a similar relationship existed. The micrographs display microcracks that were formed in the outer region of each specimen. Moreover, in the as-extruded specimen, which had few or no second-phase particles in the grains, the macrocrack propagated inward while changing its direction several times.

**Figure 5:**
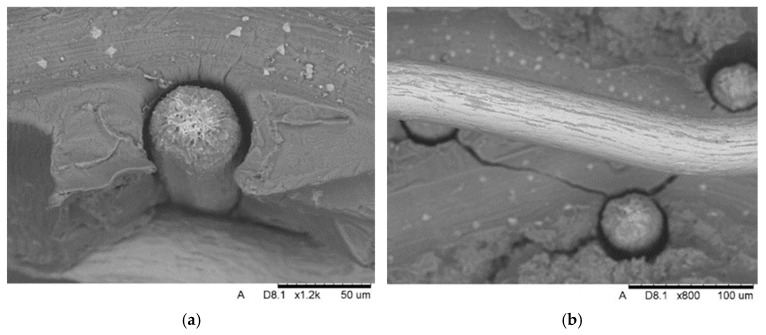
Fracture of composite with visible detachment of Ti fibre (**a**), development of microcracks between mesh layers (**b**). The scale value denoted in the figures by ‘um’ represents micrometers (µm).

Microscopic observation of fractures shows destruction of the matrix–Ti wire interface (Figure 5). The magnesium matrix is separated around the entire perimeter of the fibre, and has undergone plastic deformation with small cracks. Presumably, the interface initially breaks, and then, in a complex stress state during bending, the plastic matrix deforms significantly while the strong fibres act as reinforcing rods.

In some cases, cracks propagate to fibres lying in neighbouring mesh layers. On the one hand, this accelerates the formation of a fracture, and on the other, it stops the crack front. It would be advisable to distance neighbouring mesh layers, which would be possible at the preform preparation stage.

As mentioned the SEM microscopic observations confirmed that damage took place in the Mg-Ti beam during the three-point bending test. It was observed that the crack in the sample started at the outer edge and propagated into the sample until it reached the reinforcement, and then spread along the Ti mesh. In the study [38], the AZ91-based composite reinforced with Graphene nanoplatelets, and titanium particles was tested. In both cases, during static tensile testing, cracks formed, and when they reached the reinforcement, they propagated along it. In the case of GNPs + Ti/AZ91 samples, the crack propagated along the arranged reinforcement, but upon encountering a titanium particle, the crack changed its propagation direction. In the case of laminated composites (sandwich-type), where two types of metal alloys—AZ31 and Cu—were used, delamination was also observed after three-point bending testing, between the individual layers [39]. Furthermore, the fibres detached from the magnesium alloy due to the test, indicating a potentially weak connection at the fibre–matrix boundary. A similar problem was observed in [40], where the metal matrix composite was studied; aluminium alloy sheets were used as the matrix, while the reinforcement consisted of titanium fibres with a diameter of 1.14 mm. The strength tests included both impact testing and static tensile testing. SEM observations revealed that, in both impact-tested samples and those subjected to static tensile testing, separation occurred at the matrix–reinforcement interface. However, the mode of decohesion differed in each case. For impact-tested samples, damage at the matrix–reinforcement interface was observed only around the reinforcing fibres. In contrast, for samples subjected to static tensile testing, the authors noted that, in addition to separation at the matrix–reinforcement interface, additional cracks formed, originating in the separation zone between the matrix and the fibres. A similar situation was observed during SEM analysis of metal matrix composite samples with an AZ91 matrix reinforced with a titanium mesh. The tested samples exhibited a complex stress state. In the lower part of the sample, tensile stresses predominated, comparable to those occurring during static tensile testing. SEM observations confirmed the separation of the AZ91 matrix from the titanium fibres. Additionally, a crack propagated from the separation site, connecting to the next “separated” fibre. In another study [41,42] where the AZ91 composite reinforced with SiC particles was tested, damage to the matrix was observed very close to the reinforcement, detachment of the reinforcing particles from the matrix, and finally cracking of the composite. However, in a study where the AZ91 composite reinforced with Ti-SiC particles was tested, no detachment of the reinforcement from the matrix was observed.

Observed bright spots in the SEM investigation are intermetallic compounds of the β-Mg_17_Al_12_ phase typical for AZ91. Usually, this alloy is subjected to heat treatment, including solution and ageing, which significantly refine the precipitates, reduce eutectic regions and improve mechanical properties. In the presented investigation, solidification in a metal die, under pressure, with good contact and rapid cooling is non-equilibrium. In such conditions, the solubility of Al is reduced and the β phase precipitates can more readily form on solid Ti fibres.

**Figure 6:**
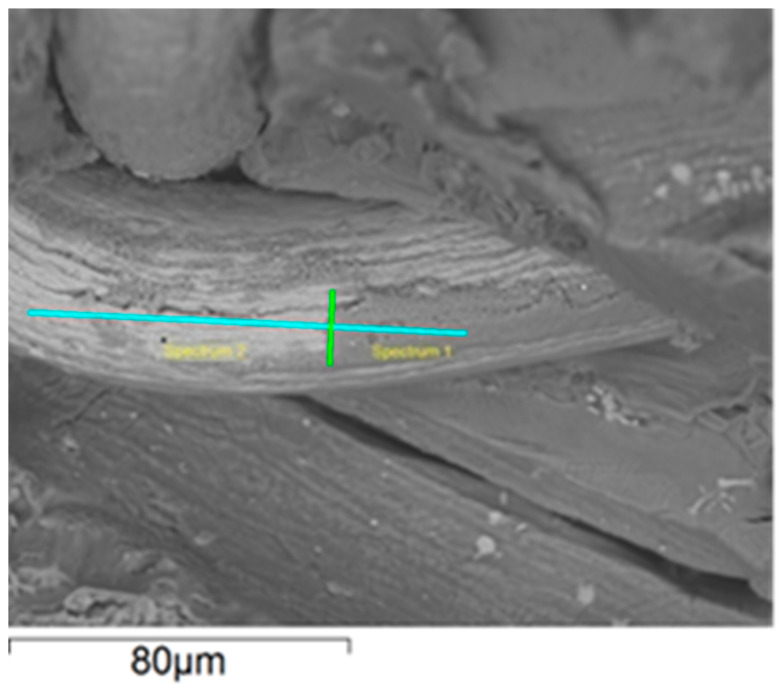
Linear EDS analysis of Ti and Mg—selected line of analyzed material. The yellow text on the illustration denotes the points ‘Spectrum 1’ and ‘Spectrum 2’ chosen for point analysis, which was performed previously supplementally in order to select the line for further analysis.

**Figure 7:**
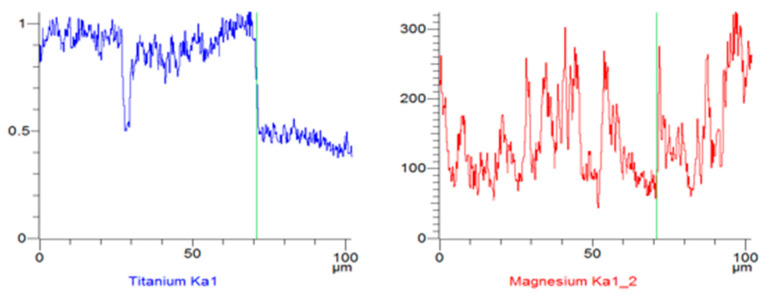
Linear EDS analysis of Ti and Mg—results.

Line-scan maps illustrated the distribution of individual composite components, namely the titanium fibres and the AZ91 alloy, after the three-point bending tests. The analyses revealed weak adhesion of the AZ91 alloy to the titanium fibres (Figure 6 and Figure 7), although isolated areas where the matrix adhered to the reinforcement were observed. From the perspective of the mechanical properties of composite materials, low or even absent adhesion between the matrix and the reinforcement can lead to a deterioration of mechanical properties.

**Figure 8:**
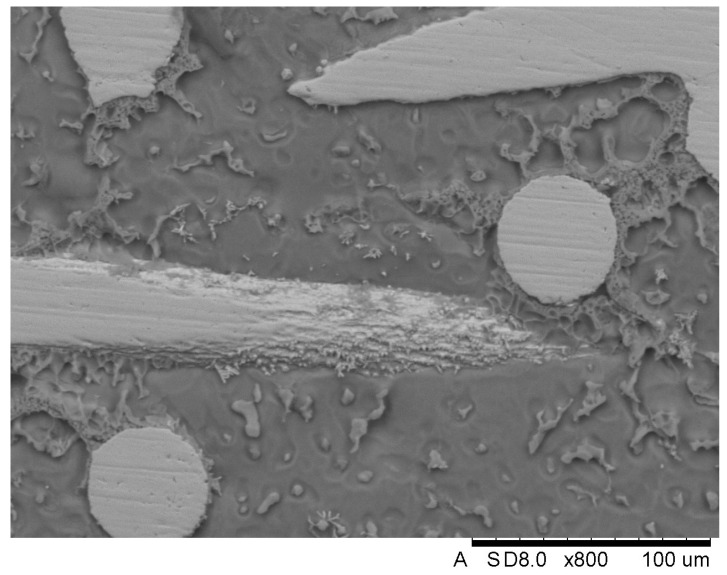
View of the surface of the sample—magnification between the fibre. The scale value denoted in the figures by ‘um’ represents micrometers (µm).

In order to explain how the introduction of mesh affected the grain size, additional SEM observations were conducted. The analyzed surface of the investigated sample presented in Figure 8 was prepared through etching in a solution of $HNO_{3}$.

Based on the visual analysis of the prepared view, it is stated that the introduction of titanium meshes did not influence the grain refinement—α phase appears similar both near the fibres and in the areas outside them. The same observation applies to the β phase. The obtained view also indicates that the β-Mg_17_Al_12_ phase exhibits a greater tendency to develop in proximity to the titanium mesh fibres.

It means that the introduction of a titanium mesh affects the improvement of mechanical properties in two aspects.

On the one hand, titanium fibres function as reinforcing rods. The present study has demonstrated that the use of titanium mesh provides benefits, mainly in improving bending strength properties. However, further mechanical testing is needed, considering tensile and compressive strength as well. The arrangement of the reinforcing mesh is crucial during tests—the direction of the mesh should be opposite to the direction of the applied force during the test.

On the other hand, as observed, the β-Mg_17_Al_12_ phase formation is somewhat more intensive in proximity to the titanium mesh fibres. It should be noted that the β-Mg_17_Al_12_ phase plays a crucial role in magnesium alloys, particularly by enhancing mechanical properties through precipitation hardening. This phase increases the tensile strength of the magnesium alloy, although it does not significantly affect its elastic properties, such as Young’s modulus.

Another key aspect that requires additional research is the adhesion between the AZ91 matrix and the Ti mesh fibres. SEM analysis revealed that, in some cases, the bond between the matrix and the reinforcement was not perfect. This could be due to a lack of adhesion between AZ91 and Ti mesh fibres during the casting process. Additionally, after conducting three-point bending tests, decohesion at the matrix–reinforcement interface was observed.

### 3.2. Aging Tests

**Figure 9:**
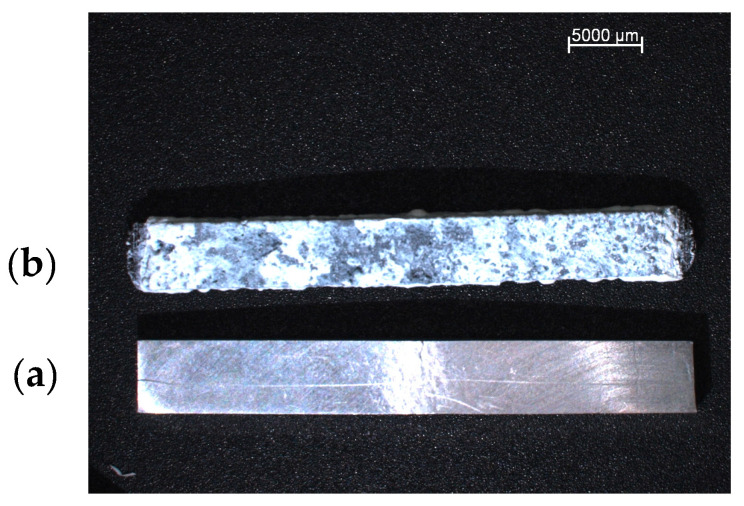
AZ91 with Ti mesh composite: (**a**)—sample before immersion in SBF solution; (**b**)—sample after 24 h of immersion in SBF solution.

**Figure 10:**
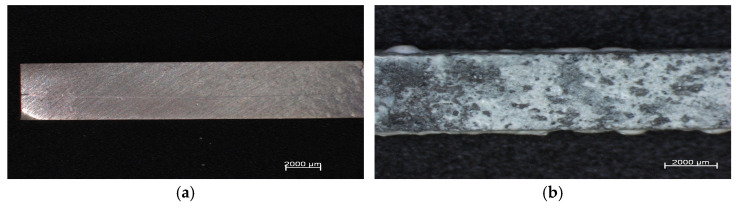
AZ91 with Ti mesh composite in magnification: (**a**)—sample before immersion in SBF solution; (**b**)—sample after 24 h of immersion in SBF solution.

The experiment involved testing the degradation of magnesium samples in SBF. The samples were weighed at specific intervals (1, 2, 4, 8, 12 and 24 h). Additionally, the pH level of the SBF was measured, along with the sample’s mass. Figure 9 and Figure 10 show the appearance of the samples before and after immersion.

**Figure 11:**
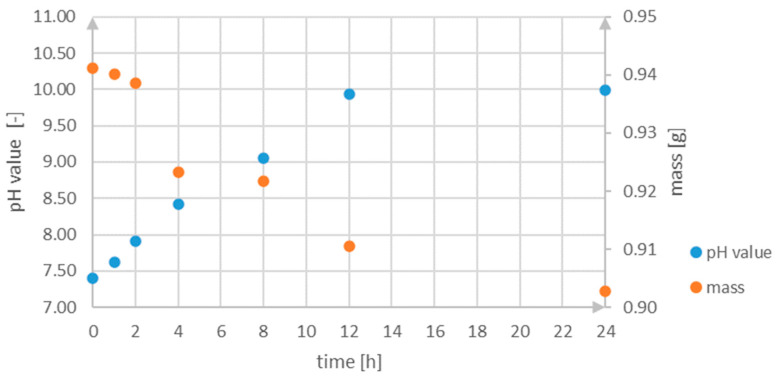
Chart providing the changes in pH value and mass during measurements.

The results shown in Figure 11 indicate that as the incubation time prolongs, the weight of the sample decreases while the pH value of the SBF rises. After the first hour, measurements already demonstrated that the pH value increased above the typical range for human physiological fluids, which is from 7.2 to 7.4 [43]. Afterwards the value of this parameter increases to 9.985 (about 10.00), characteristic of alkaline solutions. Based on this observation, it should be stated that during corrosion processes ions $OH^{-}$ form as products. The following equations present reactions that take place in the analyzed environment:

(1) $$Mg\to Mg^{2+}+2e^{-}$$

(2) $$2H_{2}O+2e^{-}\to H_{2}↑+2OH^{-}$$

(3) $$Mg^{2+}+2OH^{-}\to Mg{\left(OH\right)}_{2}$$

Similar results for AZ91 have been obtained by Xin et al. in [43]. According to their investigation, in SBF, among corrosion products (apart from hydrogen and ions $OH^{-}$), there are magnesium oxide, magnesium and calcium phosphate.

According to Figure 11, the pH value of the environment increases quickly and approximately linearly for the first 12 h, which implies a constant corrosion velocity. The result of the process is a formation of $Mg{\left(OH\right)}_{2}$. The difference between the last two measurements is not significant, which implies an equilibrium between the formation and dissolution of the corrosion products and the process was inhibited. Hence, it can be also concluded that there is a formation of an oxide layer on the sample, which protects it against further degradation. A similar nature of changes in the value of this parameter has been observed by Li et al. in [44], who comparatively investigated the corrosion properties of pure magnesium in SBF. Nevertheless, in this case (metallic Mg) the pH value stabilized after about 3 days [43,44].

The chemical reaction equations presented above take place first of all in an aqueous environment. However, the SBF solution contains chloride, sulfate, carbonate, and phosphate ions, which make the environment aggressive in terms of corrosion. The forming magnesium hydroxide $Mg{\left(OH\right)}_{2}$, which is sparingly soluble in water, reacts with chloride ions to magnesium chloride $Mg{Cl}_{2}$, which is readily soluble. It implies that the surface becomes more active or decreases the protected area, which, as a result, promotes further dissolution of magnesium. This reaction can be presented as follows:

(4) $$Mg{\left(OH\right)}_{2}+2{Cl}^{-}\to {MgCl}_{2}+2{OH}^{-}$$

Moreover, Song et al. [45] also suspect that chloride ions accelerate intermediate electrochemical reactions, which transform magnesium into magnesium univalent ions.

The velocity of corrosion processes in this environment is also enhanced by anions $HCO_{3}^{-}$. Pébère et al. [46] indicate that the corrosion rate increases when their concentration is greater than about 40 $\frac{mg}{dm^{3}}$ due to accelerated dissolution of the $Mg{\left(OH\right)}_{2}\;(MgO)$ protection layer. And it should be noted that the $HCO_{3}^{-}$ concentration in the SBF is so much higher and equals approximately 256 $\frac{mg}{dm^{3}}$ [43].

It should be noted that only one sample was investigated in the conducted ageing test. Before mass measurement, the sample was pulled from the SBF solution and afterward dried. It was not cleaned with any chemical substance to remove corrosion products. In the next step, the sample was weighed and then immersed again in the same SBF environment. Due to the above reason, the obtained results of mass change in the analyzed time range are only indicative and cannot be the basis for determining the corrosion rate.

Due to the lower protection of formed products, corrosion is most intense in the initial phase after immersion. Afterward, passivation of the active surface and accumulation of corrosion products decrease the corrosion rate. After a sufficiently extended immersion, a balance is reached between the creation and dissolution of the corrosion products, resulting in steady degradation rates [43].

**Figure 12:**
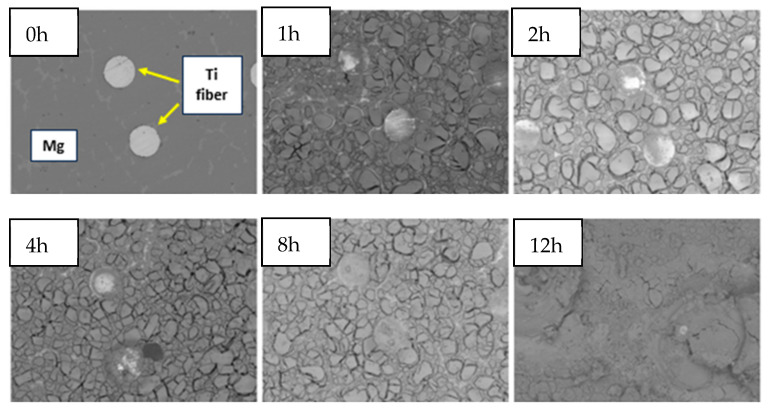
SEM pictures of AZ91 with Ti mesh composite surface before and after immersion for 1, 2, 4, 8 and 12 h in SBF. The Figures performed in the same magnification.

The surface structure of the tested samples following immersion in the SBF solution was determined using SEM. Figure 12 shows the surface of AZ91–Ti mesh composite with titanium fibres, before and after immersion.

Even though the total immersion time was 24 h, SEM images were only for up to 12 h of immersion. Due to the formation of a layer of oxides, further analysis and location of titanium fibres were impossible. These findings are not consistent with the previous literature. In a study by Ocal et al. [47], the degradation of AZ91 in SBF was determined. During incubation for specific time periods (1, 2, 4, 8 and 24 h), a corrosion layer of oxides was observed, as well as volcanic structures (such as craters) forming on the sample surface. The number of these structures increased with longer immersion times. However, after 24 h of immersion, the volcano structures were destroyed and surrounded by a thick corrosion product rich in Ca, P and O. In our composite samples, the volcanic structure was only observed after 24 h of immersion in SBF (Figure 12).

### 3.3. Microtomographic Analysis

Micro-computed tomography, as a non-destructive testing method, enables the observation of sample changes associated with its degradation in an SBF solution. One of its greatest advantages is its volumetric nature, which allows for high-resolution analysis of changes not only on the surface but throughout the entire volume of the examined sample.

**Figure 13:**
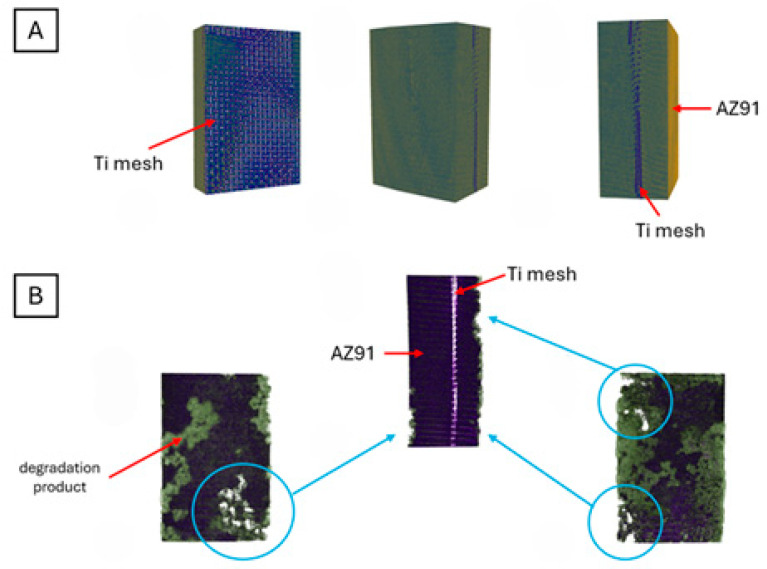
Microtomographic reconstructions of the AZ91 with Ti mesh composite before (**A**) and after (**B**) immersion in SBF fluid; view of Ti mesh as a reinforcement of the composite based on AZ91.

In the presented work, 3D reconstructions were performed for samples both before and after degradation in SBF fluid. A 3D reconstruction of the samples before degradation allows for determining how the reinforcement is distributed within the magnesium matrix, which may have shifted during the casting process (Figure 13A). After degradation in SBF fluid, the 3D reconstructions revealed pitting corrosion that formed on the surface of the sample (Figure 13B) and the layer of degradation product (Figure 13B, green colour). Similar results were obtained by Li et al. [26], who analysed the degradation process of magnesium scaffold (AZ91 alloy) in SBF. They measured pore size, sample volume and porosity using microtomography. Additionally, they presented the distribution of degradation products in each plane of the sample on 3D reconstructions. Pitting corrosion is the second most common type of corrosion of magnesium and its alloys. It usually occurs locally on the surface of the alloy, particularly Mg-Al [45]. When immersed in a water environment with corrosive ions, shallow pits form on the magnesium surface, resulting from the breakdown of the protective passive layer. In the case of magnesium alloys, microgalvanic cells are formed around impurities or secondary phases, causing corrosion to spread along the magnesium matrix surrounding particles, thereby increasing the volume of the pit until a new passive corrosion product $Mg{\left(OH\right)}_{2}$ is precipitated [48].

## 4. Conclusions

The aim of this work was to prepare magnesium matrix composites with titanium mesh (AZ91 with two layers and AZ91 with three layers) via the squeeze casting method and to determine their microstructure, mechanical properties and behaviour during strength tests. To achieve this, a scanning electron microscope, microtomography and three-point bending tests were used. Additionally, accelerated ageing tests were conducted to determine the behaviour of the samples in SBF. Based on the results obtained, the following conclusions can be drawn:

- Three-point bending tests showed that with an increased amount of titanium mesh in the composite, the bending strength increased. However, for Young’s modulus, a decrease in its value was observed for three layers of titanium mesh.
- SEM observations showed that, as a result of strength tests, titanium fibres detach from the magnesium matrix. Cracks that appear during three-point bending propagate from the edge of the sample and then along the placed reinforcement.
- Accelerated ageing tests showed that the pH of the SBF increases approximately linearly to a value around 10.00, which investigates the formation of $Mg{\left(OH\right)}_{2}$. Afterward, this value stabilised, which implies the formation of an oxide layer on the sample and a state of dynamic equilibrium. Amidst corrosion products, there are ions of $OH^{-}$, hydrogen gas and compounds containing magnesium, oxygen, calcium and phosphorus.
- Microtomography is a process that enables us to determine the internal structure of the samples without damaging them. In the presented 3D reconstructions of the samples following immersion in SBF, darker areas can be observed, which indicates the formation of degradation products in the sample. However, by using a 3D reconstruction processing program, it was possible to look inside the sample to check whether the SBF also affected the titanium mesh. Therefore, it is recommended that this method be used for further research.

## Figures and Tables

**Table 1:** Chemical composition of AZ91 magnesium alloy.

Element	Mg	Al	Zn	Mn	Si
**wt.%**	89.8	9.00	1.00	0.13	0.05

**Table 2:** Chemical composition of titanium mesh—Titanium gauze.

Element	C	Fe	H	N	O	Ti
**wt.%**	0.008–0.016%	0.03–0.04%	0.002–0.003%	0.004%	0.048–0.05%	99.897–99.898%

**Table 3:** Composition of human blood and SBF [33,34].

Ion	Simulated Body Fluid (SBF) [mM]	Blood Plasma [mM]
$Na^{+}$	142.0	142.0
$K^{+}$	5.0	5.0
$Mg^{2+}$	1.5	1.5
$Ca^{2+}$	2.5	2.5
$Cl^{-}$	147.8	103
$HCO_{3}^{-}$	4.2	27.0
$HPO_{4}^{2-}$	1.0	1.0
$SO_{4}^{2-}$	0.5	0.5

**Table 4:** Microtomography parameters of the tested samples.

Parameter	Parameter Value
rotation step	0.2 deg
exposure time	2100 ms
charging current	112 µA
voltage	89 kV
pixel size	3 µm
filter	Al + Cu

**Table 5:** Results of three-point bending test: AZ91, AZ91 + two layers of Ti mesh and AZ91 + three layers of Ti mesh.

Material	Flexural Strength [MPa]	E [GPa]	Break Strain [%]
AZ91	242.5 ± 7.5	25 ± 2	2.7 ± 0.2
AZ91 + 2 mesh layers	270 ± 13	27.5 ± 0.5	3.45 ± 0.35
AZ91 + 3 mesh layers	295 ± 10	23.5 ± 0.5	4.95 ± 0.55

## Data Availability

The original contributions presented in this study are included in the article. Further inquiries can be directed to the corresponding author.
